# Early and Sustained Clinical Benefits of Benralizumab in Severe Eosinophilic Asthma: Findings from the ORBE II Study

**DOI:** 10.3390/jcm14093011

**Published:** 2025-04-26

**Authors:** Pilar Ausín, María Eugenia Navarrete-Rouco, Luis Carazo, Jose Luis Sanchez-Trincado, Elisa Luzon, Javier Nuevo, Mónica Santín, Jesús Sánchez, Alicia Padilla-Galo

**Affiliations:** 1 Pulmonology Department, Hospital del Mar, 08003 Barcelona, Spain; 2 Pharmacy Department, Hospital del Mar, 08003 Barcelona, Spain; 3 Pulmonology Department, Complejo Asistencial Universitario de León, 24071 León, Spain; lcarazo@saludcastillayleon.es; 4 Medical Department, AstraZeneca Farmacéutica S.A., 28050 Madrid, Spainjavier.nuevo@astrazeneca.com (J.N.);; 5 Pulmonology Department, Hospital Universitario Virgen de la Victoria, 29010 Málaga, Spain

**Keywords:** benralizumab, severe eosinophilic asthma, ORBE II, real world, early response, super-response, ACT, FEV_1_, clinical remission

## Abstract

**Introduction:** Benralizumab has demonstrated rapid efficacy in treating severe eosinophilic asthma (SEA). This study aims to characterize early responses to benralizumab, the patient features observed in those with early responses, and the potential patient features that could predict them, and it also evaluates whether these improvements are sustained during a one-year follow-up (FUP) in clinical practice. **Methods:** This analysis was conducted using the ORBE II study database. ORBE II is an observational, retrospective study that included uncontrolled SEA adult patients treated with benralizumab according to routine clinical practice in Spain. We analysed patients with available data on the asthma control test (ACT) at baseline and within the first 120 days after benralizumab initiation, identifying ACT “Early Super-Responders” (ACT-ESR) as patients with a ≥9 point-improvement in the ACT score or reaching an absolute score of ≥24. Likewise, we assessed patients with available data on the pre-BD FEV_1_ during the same study periods, defining those with a pre-BD FEV_1_ increment of ≥230 mL as FEV_1_-ESR patients. Clinical outcomes were described up to 1 year of FUP. **Results:** A total of 45 and 65 patients with data for ACT and FEV_1_, respectively, during the first 120 days of treatment were analysed. Of those, 55.5% and 58.5% of patients were categorized as ACT-ESR and FEV_1_-ESR, respectively. At baseline, both groups showed high T2 inflammation markers and a high prevalence of comorbidities (chronic rhinosinusitis with nasal polyposis: 56% and 50%; gastro-oesophageal reflux disease: 24% and 40%, respectively). Poor asthma control (ACT < 20) was observed at baseline in 96% of ACT-ESR, while impaired lung function (pre-BD FEV_1_ < 80%) was present in 71.7% of FEV_1_-ESR. Oral corticosteroid (OCS) dependency affected 25% and 30% of ACT-ESR and FEV_1_-ESR, respectively. The early gains observed in ACT-ESR and FEV_1_-ESR were sustained up to 1 year of FUP, with 90.5% and 66.7% of patients achieving a super-response (zero exacerbations and no OCS use) and 92.0% and 71.1% meeting clinical remission criteria (zero exacerbations, no OCS use, ACT ≥ 20 and pre-BD FEV_1_ increment of ≥100 mL), respectively. **Conclusions:** Benralizumab provides early benefits for SEA patients in clinical practice, with more than half achieving early super-responses in both ACT score and lung function. These improvements were sustained over a 1-year FUP, resulting in high rates of clinical remission.

## 1. Introduction

Benralizumab, a humanized monoclonal antibody targeting the interleukin-5 receptor α subunit (IL-5Rα), has emerged as an advanced add-on therapeutic agent for patients with severe eosinophilic asthma (SEA). Pivotal clinical trials showed its efficacy in reducing the frequency of severe exacerbations (SE) and use of maintenance oral corticosteroids (mOCS), as well as improving asthma control and lung function [1,2,3]. Its mechanism of action rapidly depletes blood and sputum eosinophils to nearly undetectable levels [4,5]. However, the effects of benralizumab extend beyond eosinophils, as other immune cells such as basophils and type 2 innate lymphoid cells (ILC2) might be also reduced [6,7,8], further dampening several inflammatory pathways [9].

Mounting evidence has suggested that benralizumab has the potential to promote a rapid clinical response. For instance, the SIROCCO and CALIMA trials reported symptomatic relief as early as 3 days post-benralizumab initiation [10,11]. Other clinical studies have found significant improvements in patient-reported outcomes (PROs), asthma control, and lung function as early as 2 to 4 weeks after initiating benralizumab [12,13]. Notably, clinically relevant improvements in nasal polyposis (NP) symptoms were sustained from week 4 onwards in SEA patients with comorbid NP [12]. Beyond the controlled setting of randomized clinical trials (RCTs), several real-world studies from the international XALOC programme have documented discernible early benefits in patient-reported outcomes (PROs) and asthma control, supporting the rapid effect of benralizumab [14,15,16]. In a cohort of SEA patients, 35% achieved clinical remission at 6 months, defined as no exacerbations, no maintenance oral corticosteroid (OCS) use, asthma symptom control (asthma control test [ACT] score ≥ 20), and no decrease in the forced expiratory volume in 1 s (FEV_1_) ≥ 200 mL [17]. However, there is little information regarding the characterization of patients who achieve an early super-response, or whether an early response to benralizumab is associated with sustained long-term benefits. In this study, using the real-world ORBE II cohort of SEA patients [18], we sought to describe the baseline clinical features of early super-responders (ESR) and to assess whether early treatment outcomes were associated with sustained benefits over time.

## 2. Methods

### 2.1. Study Design

ORBE II (NCT04648839) was an observational study conducted across multiple centres in Spain, retrospectively assessing adult patients (≥18 years) with uncontrolled SEA treated with benralizumab in routine clinical settings after its commercialization [18]. ORBE II investigators and sites are detailed in Appendix A. In this subgroup analysis, patients from the ORBE II cohort were classified as “Non-Early Responders” (NER), “Early Responders” (ER), or “Early Super-Responders (ESR)” based on changes in ACT scores and pre-bronchodilator FEV_1_ (pre-BD FEV_1_) values from baseline to 120 days post-benralizumab initiation. This 120-day period was selected considering the retrospective nature of the study and to ensure sufficient clinical data availability. Patients from the ORBE II database with no available ACT or FEV_1_ data during this period were not considered for this analysis.

Baseline clinical and sociodemographic characteristics of these patient subgroups were described over the 12 months preceding the index date (i.e., benralizumab initiation), and outcomes were collected throughout a 1-year follow-up (FUP) period. For non-continuous variables (such as ACT and FEV_1_), baseline measurements correspond to the closest recorded measurement prior to benralizumab initiation.

### 2.2. Classification of Patients Based on ACT Early Response

The ACT score values at baseline and within the first 120 days after treatment initiation were selected. From the 120 days of FUP values, only the highest one was subsequently considered for the analysis. Patients were then classified considering both this highest ACT score achieved within the 120 days post-benralizumab initiation and the difference between this value and the baseline, as follows:

- Non-Early Responders (NER): Patients with an ACT difference < 3 points.
- Early Responders (ER): Patients with an ACT difference of ≥3 points but <9 points, and an absolute score < 24.
- Early Super-Responders (ESR): Patients with an ACT difference of ≥9 points or an absolute score ≥ 24.

ACT cut-off values for the identification of early responses were defined by the authors based on their clinical opinion. Accordingly, an early super-response was defined as 3 times the minimal clinically important difference (increase of 3 points) or patients with values close to the maximum score. Patients with a baseline ACT ≥ 24 were excluded from the analysis as they cannot further respond in relation to this parameter.

### 2.3. Classification of Patients Based on FEV_1_ Early Response

Pre-BD FEV_1_ values at baseline and within the first 120 days after treatment initiation were selected. From the 120 days of FUP values, only the highest one was subsequently considered for the analysis. Patients were then classified considering both this highest pre-BD FEV_1_ value achieved within the 120 days post-benralizumab initiation and the difference between this value and the baseline, as follows:

- Non-Early Responders (NER): Patients with a pre-BD FEV_1_ difference of <100 mL.
- Early Responders (ER): Patients with a pre-BD FEV_1_ difference of ≥100 mL but <230 mL.
- Early Super-Responders (ESR): Patients with a pre-BD FEV_1_ difference of ≥230 mL.

Cut-off values for the FEV_1_ increment (≥100 and ≥230 mL) to identify early responses were selected by the authors based on their clinical opinion and as published in previous studies [18,19].

### 2.4. Statistical Analysis

The analysis of sociodemographic and clinical characteristics in the predefined patient subgroups was carried out using the same methodological approach as in the overall ORBE II cohort [18]. Specifically, for continuous variables, descriptive statistics included the number of valid cases, mean, standard deviation (SD), and, when appropriate, the median and interquartile range (Q1–Q3). Categorical variables were summarised as absolute frequencies and percentages. Consistent with the retrospective observational design of the original study, the current analysis was descriptive in nature; thus, no inferential statistics (e.g., *p*-values) or multivariate models were applied, as the design and sample size did not support such analyses. All statistical procedures were performed using R software (version 4.3.0; www.R-project.org), along with the “dplyr”, “gtsummary”, and “psych” packages.

## 3. Results

### 3.1. Distribution of Patients Based on ACT or Pre-BD FEV_1_ Responses to Benralizumab from Day 0 to 120

**Figure 1:**
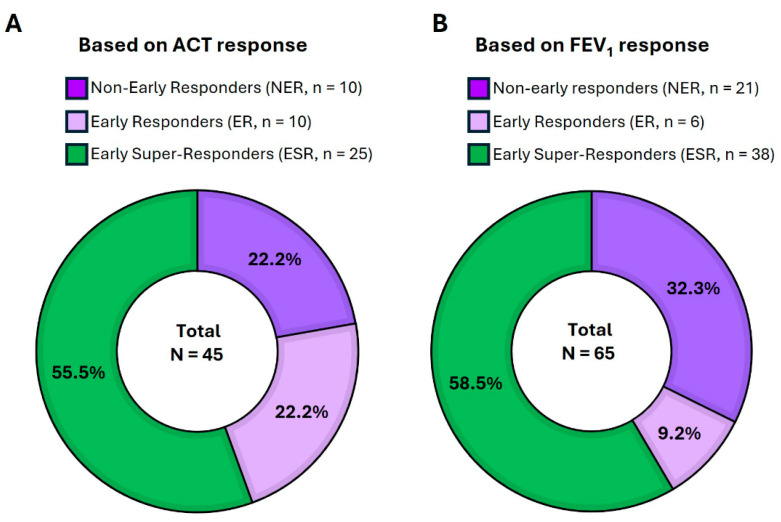
**Classification of patients with evaluable ACT and FEV_1_ data according to their response during the first 120 days after initiating benralizumab**. (**A**) The stratification of patients with available ACT data based on the observed change in ACT score achieved from baseline to 120 days under benralizumab. Based on the established criteria, patients showing an ACT score difference < 3 points were considered non-early responders (NER); patients with an ACT difference of ≥3 points, but <9 points, and a final follow-up ACT score < 24 were considered early responders (ER); and patients with an ACT difference of ≥9 points or a final follow-up ACT score ≥ 24 were considered early super-responders (ESR). (**B**) The distribution of patients with available data on pre-BD FEV_1_ according to the response achieved from baseline to 120 days following benralizumab initiation in terms of pre-BD FEV_1_ gain (mL). Based on the pre-specified criteria, patients were classified as follows: NER, when the pre-BD FEV_1_ difference between baseline and the highest follow-up value was ≤100 mL; ER when the pre-BD FEV_1_ difference was ≥100 mL but <230 mL; and ESR when the pre-BD FEV_1_ difference was ≥230 mL. ACT, asthma control test; BD, bronchodilator; FEV_1_, forced expiratory volume in 1 s.

The total population (TP) for each analysis was defined separately. The TP for ACT score (ACT-TP) comprised all patients for whom ACT values were available both at treatment initiation and within the first 120 days of follow-up (N = 45, excluding five patients who had already shown an ACT score ≥ 24 at baseline); the TP for pre-BD FEV_1_ (FEV_1_-TP) included patients with documented pre-BD FEV_1_ measurements at baseline and within the initial 120-day follow-up window (N = 65). While 10 (22.2%) and 6 (9.2%) patients from each of the TPs reached the criteria for ACT or pre-BD FEV_1_ early responders (ER), respectively, we found that more than half of the patients from both TPs met the established definition for ESR in each separate analysis, with 25 (55.5%) for ACT (ACT-ESR) and 38 (58.5%) for pre-BD FEV_1_ (FEV_1_-ESR) (Figure 1A,B).

The number of patients who simultaneously met both ACT-ESR and FEV_1_-ESR criteria, considering only patients with available data for both parameters at baseline and between 0 and 120 days (n = 24), was evaluated. Out of these 24 patients, 15 (62.5%) could be considered both ACT-ESR and FEV_1_-ESR.

### 3.2. Baseline Characterization of ACT-ESR

At baseline, the ACT-ESR patient subgroup showed particularly high levels of T2 inflammation markers, with a median (interquartile range [IQR]) blood eosinophil count (BEC) of 650.0 [440.0–1030.0] cells/µL and a median [IQR] fractional exhaled nitric oxide (FeNO) of 46.0 [18.3–100.0] ppb. Median IgE values were similar to those found in the ACT-TP (Table 1). Furthermore, a higher prevalence of comorbid chronic rhinosinusitis with nasal polyps (CRSwNP) (56.0%) and gastroesophageal reflux disease (GERD) (24.0%) was found among ACT-ESR.

In the year preceding benralizumab treatment, ACT-ESR patients had a mean (SD) of 2.5 (1.3) severe exacerbations, which was similar to that observed in the ACT-TP (Table 2). Asthma control was poor overall, particularly among ACT-ESR, with 96.0% of patients having an ACT score < 20. Lung function was also especially impaired at baseline in ACT-ESR patients, considering that 80.0% of them had a pre-BD FEV_1_ < 80% predicted. Regarding OCS use, similar proportions of patients classified as corticosteroid-dependent (i.e., those who had received mOCS for ≥3 months during the year preceding benralizumab initiation) were observed in the ACT-TP and ACT-ESR groups (27.3% for the ACT-TP and 25.0% for ACT-ESR); however, ACT-ESR patients used almost half the median mOCS daily dose (Table 2).

### 3.3. Baseline Characterization of FEV_1_-ESR

When analysing both the FEV_1_-TP and the FEV_1_-ESR subgroups, similar BECs were observed, although they were particularly elevated in the FEV_1_-ESR subgroup, with a median (IQR) of 550 (300.0–900.0) cells/µL. FeNO and IgE concentrations were similar between subgroups, with FEV_1_-ESR having a median (IQR) of 30.0 (19.0, 65.5) ppb and 97.0 (47.0, 310.0) IU/mL of these biomarkers, respectively. Almost all FEV_1_-ESR patients had comorbidities (97.4%), showing a 50.0% and almost 40% prevalence of comorbid CRSwNP and GERD, respectively (Table 2). Both FEV_1_-TP and FEV_1_-ESR subgroups showed a similarly elevated mean number of severe exacerbations during the 12 months prior to benralizumab initiation (Table 2). Overall, FEV_1_-ESR patients had slightly worse asthma control and lung function, with 85.2% of them having an ACT score <20 and 71.1% with a pre-BD FEV_1_ <80% predicted (Table 2).

### 3.4. Temporal Dynamics of ACT Score and Pre-BD FEV_1_ Values During Benralizumab Treatment

**Figure 2:**
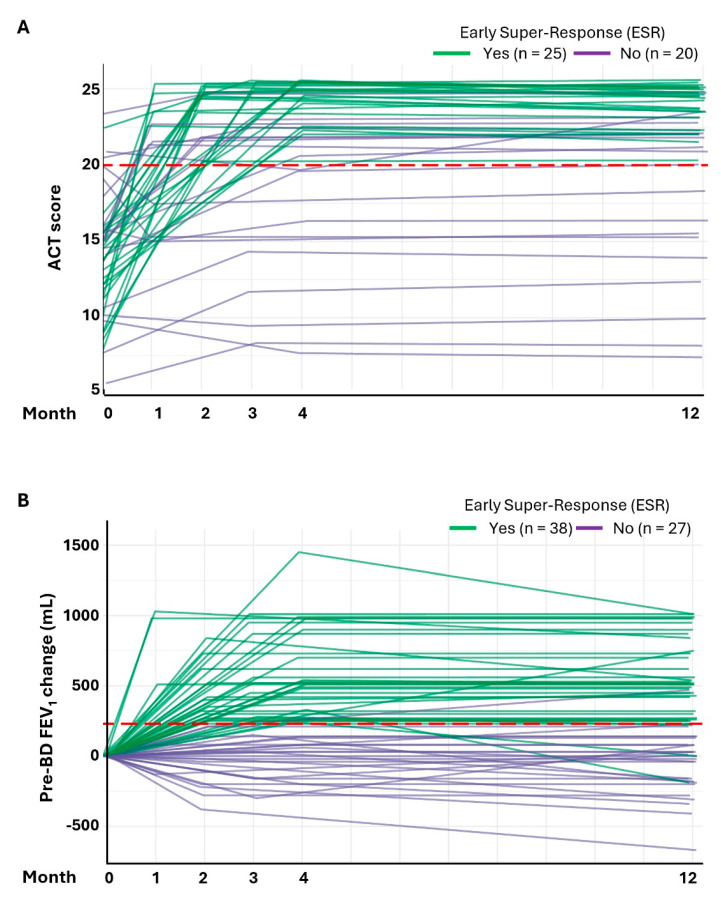
**Temporal dynamics of ACT score and pre-BD FEV_1_ change during benralizumab treatment in patient subgroups.** (**A**) Trajectory of ACT scores over a 12-month period in ACT scores discriminating between ACT-ESR patients (n = 20) and those who did not achieve an ACT early super-response (NER + ER; n = 20). (**B**) The graph plots pre-BD FEV_1_ changes over a 12-month period, discriminating between FEV_1_-ESR patients (n = 38) and those who did not achieve an FEV_1_ early super-response (NER + ER; n = 27)). Each line represents an individual patient. The dashed red lines represent the following clinically relevant thresholds: an ACT score of 20 (Panel A) and a pre-BD FEV_1_ increment of 230 mL (Panel B). ACT, asthma control test; BD, bronchodilator; FEV_1_, forced expiratory volume in 1 s; NER, non-early responders; ER, early responders; ESR, early super-responders.

We monitored individual ACT score changes and lung function (FEV_1_) improvements at several time points during the 12-month follow-up period, discriminating between ESR patients and those who did not achieve an early super-response (both NER and ER). At baseline, ACT-ESR patients (n = 25) presented a relatively homogenous range of initial ACT scores, predominantly clustered between 10 and 15 (mean [SD] ACT score of 13.0 [3.2], Table 2), which was markedly different to that observed in the rest of the patients (Figure 2A). The differentiation in the ACT score trajectory between these subgroups became evident early during the first 4 months and was sustained over the course of the FUP. Indeed, as early as month 1 following benralizumab initiation, the ACT-ESR subgroup nearly doubled the mean ACT score recorded at baseline, with a mean (SD) ACT score of 24.1 (1.6), maintaining this improvement throughout the monitoring period. In the case of those not achieving an ACT early super-response (n = 20), 70% also showed a modest improvement in asthma symptom control during the first 120 days, which was generally maintained thereafter, improving by nearly 3 points (from 15.05 to 18.0) by the end of the FUP period.

A clear divergence in the trajectory of the pre-BD FEV_1_ increment between FEV_1_-ESR patients and those that did not reach an FEV_1_ early super-response began to be evident from the first month post-treatment initiation (Figure 2B). At 120 days, the FEV_1_-ESR subgroup benefited from a marked pre-BD FEV_1_ gain that reached a clinically meaningful increment of ≥500 mL in 21 (55.3%) of these patients. The remarkable beneficial action of benralizumab observed among FEV_1_-ESR during the first 4 months was sustained over the 1-year FUP period (Figure 2B).

### 3.5. Clinical Outcomes at 1-Year Follow-Up

#### 3.5.1. ACT-ESR

**Figure 3:**
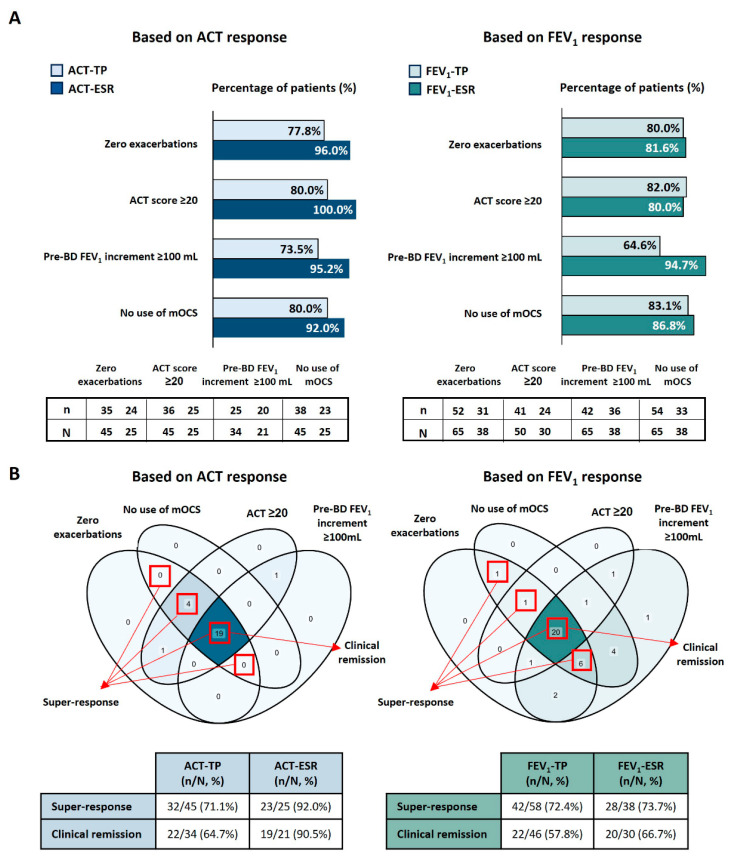
**Attainment of clinical outcomes after 1-year follow-up with benralizumab treatment.** (**A**) Proportion of patients who met pre-defined clinical objectives in response to benralizumab in the ACT-TP and ACT-ESR subgroups (left graph) and in the FEV_1_-TP and FEV_1_-ESR subgroups (right graph). (**B**) The Venn diagrams show the number of ESR achieving one or more of the four clinical objectives (zero exacerbations, no use of mOCS, ACT score ≥ 20, and pre-BD FEV_1_ increment ≥100 mL). The left diagram represents the ACT-ESR subgroup, while the right diagram represents the FEV_1_-ESR group. The tables below each diagram show the proportion of patients that achieved super-response and clinical remission. Percentages were calculated based on the number of patients within each subgroup who had available data for the parameter or a combination of parameters under analysis (N). The count of patients who reached each clinical goal or response threshold to benralizumab is indicated as “n”. ACT, asthma control test; BD, bronchodilator; BEC, blood eosinophil count; FeNO, fractional exhaled nitric oxide; FEV_1_, forced expiratory volume in 1 s; mOCS, maintenance oral corticosteroids; ppb, parts per billion; ESR, early super-responders; TP, total population.

In general, all patients in the ACT-TP showed substantial clinical improvements at 1-year FUP with benralizumab treatment in parameters such as exacerbations, OCS use, or lung function. However, the ACT-ESR subgroup particularly benefited, with a remarkable 96.0% of patients reporting zero severe exacerbations (Figure 3A) and an overall 96.0% reduction in severe exacerbations (Table 2). Additionally, a 100% reduction in hospitalizations and emergency department (ED) visits was confirmed in both the ACT-TP and ACT-ESR subgroups.

Asthma control was substantially improved in the ACT-ESR subgroup, with 100% of patients showing a rise in the ACT score ≥ 3 points (with a mean [SD] increase of 11.1 [3.1] points) and an ACT score ≥ 20 (Table 2; Figure 3A). ACT-ESR patients also showed a marked improvement in lung function, with a mean (SD) gain in pre-BD FEV_1_ of 620.0 (327.2) mL, with 95.2% of them experiencing a ≥100 mL increment in pre-BD FEV_1_. Although the percentage of corticosteroid-dependent patients in the ACT-TP decreased from 27.3% at baseline to 15.6% at the 1-year FUP and the median daily mOCS dose was reduced by 76.9%, better outcomes were observed in the ACT-ESR subgroup. Specifically, these patients experienced a 100% reduction in the median mOCS dose, resulting in 92.0% of them showing no use of mOCS at 1-year FUP (Table 2; Figure 3A). Finally, while around 71% and 64% of the ACT-TP achieved a super-response to benralizumab (defined as zero exacerbations and no use of mOCS) and clinical remission (defined as zero exacerbations, no use of mOCS, ACT score ≥ 20, and pre-BD FEV_1_ improvement of ≥100 mL), respectively, almost the entire ACT-ESR subpopulation (>90%) met the criteria for clinical remission (Table 2; Figure 3B).

#### 3.5.2. FEV_1_-ESR

After 1-year FUP, a drastic reduction in severe exacerbations, hospitalizations, and ED visits was similarly observed in both FEV_1_-TP and FEV_1_-ESR populations. For instance, severe exacerbations were reduced by more than 85%, and ≥80% of all evaluated patients achieved total SE eradication (Table 2; Figure 3A). Regarding asthma symptom control, 79.1% of the FEV_1_-TP achieved a clinically relevant increase in the ACT score (≥3 points; mean [SD]: 8.1 [5.6]), while a higher proportion of FEV_1_-ESR reached this threshold (88.5%; mean [SD]: 9.4 [5.7]). In addition, FEV_1_-ESR remarkably improved their lung function, with a mean (SD) pre-BD FEV_1_ increment of 537.4 (295.0) mL and almost 95% of patients exhibiting a clinically relevant increment of ≥100 mL. The median daily dose of mOCS was reduced by 100% in both populations. However, up to 80% of FEV_1_-ESR achieved complete mOCS withdrawal, and 86.8% of patients did not use mOCS at the end of FUP (Table 2; Figure 3B). Finally, while a super-response was similarly achieved in both FEV_1_-TP and FEV_1_-ESR populations by 72–74% of patients, a higher proportion of FEV_1_-ESR (66.7% vs. 57.8% in the FEV_1_-TP) achieved clinical remission (Figure 3B).

## 4. Discussion

The clinical benefits of benralizumab in the treatment of SEA have been extensively documented in clinical trials and real-world studies [20,21,22,23,24,25], including the ORBE II study [18]. In this subgroup analysis of the ORBE II study database, we provide further specific evidence on the early and sustained effectiveness of benralizumab, consistent with reported real-world data on its beneficial effects on asthma control, lung function, and PROs [14,15,16,17,23,26].

The results of our study showed that the effectiveness of benralizumab becomes quickly apparent after initiating treatment in more than half of the SEA patients with available data on ACT or pre-BD-FEV_1_ values during the first 120 days of FUP within the ORBE II cohort. These patients were considered ESR, achieving more than minimally clinical important differences in the ACT score and pre-BD-FEV_1_ values. Our findings are in line with prior evidence showing early responses to benralizumab, even at earlier time points. Post hoc analysis of the CALIMA and SIROCCO clinical studies of benralizumab confirmed lung function improvements as early as the first week following treatment initiation [10]. Also in relation to asthma control, a study conducted by Noorduyn et al. (2024) showed a mean (95% CI) overall change in the ACQ-6 score at week 1 of −0.82 (−0.99, −0.65; n = 127) and confirmed a continuous improvement over the first 24 weeks of follow-up [15].

The baseline characterization of the ACT-ESR and FEV_1_-ESR subgroups revealed some distinctive features of early super-responders, such as particularly elevated BEC or high FeNO in the case of ACT-ESR. Our characterization of ESR patients provides additional evidence supporting the role of elevated T2 airway inflammation biomarkers (such as high BEC and FeNO) as well as the presence of T2-related comorbidities, particularly CRSwNP, as factors commonly observed in patients with early and sustained responses to benralizumab [18,27,28,29,30,31]. In this context, this information may be valuable for clinicians to guide their decision-making in routine clinical practice.

The serial monitoring of ACT and pre-BD FEV_1_ over 1-year FUP clearly revealed substantial and early improvements in asthma control and lung function among ESR, with remarkable gains observed during the first month after benralizumab initiation. These findings on the early effectiveness of benralizumab are in line with prior reports supporting its potential role as a rapid rescue therapy for patients with severe asthma attacks. Notably, a single dose of benralizumab has been shown to significantly reduce exacerbation recurrences and even provide marked improvements in lung function within 24 h [32,33,34]. In this context, the ABRA study has recently shown that benralizumab can be used as a treatment for acute eosinophilic exacerbations in asthma and chronic obstructive pulmonary disease (COPD), achieving better results than prednisolone alone as the standard of care [35]. Furthermore, these early improvements were sustained throughout the evaluation period, resulting in high rates of super-response and clinical remission as defined by published criteria [28,36,37,38] after 1-year FUP, particularly among ACT-ESR patients, suggesting that an improvement in ACT may be a more reliable indicator of clinical response than improvement in FEV_1_. These sustained long-term benefits are consistent with previous reports indicating that these effects persist or even improve over time, with responses at 4 weeks serving as predictors of outcomes for up to 4 years [23,37,39,40]. Sustained benefits in the reduction of exacerbations and improved asthma control have also been linked in several studies to higher persistence to benralizumab treatment [18,23,31]. Importantly, we observed that SEA patients without an early response to benralizumab could still achieve clinical benefits later during the 1-year FUP period, emphasizing the importance of maintaining benralizumab treatment in these patients. This is consistent with findings by Jackson et al. (2022), who reported that a lack of early response at 4 weeks did not predict the absence of meaningful outcomes at 48 weeks (negative predictive value, 22.4%) [37].

Beyond asthma, benralizumab has demonstrated rapid benefits in other chronic conditions, such as eosinophilic granulomatosis with polyangiitis (EGPA) and CRSwNP. In the MANDARA trial, which compared benralizumab to mepolizumab for EGPA, benralizumab led to a more pronounced reduction in BEC as early as 1 week after initiation, with nearly 40% of benralizumab-treated patients achieving remission by week 16 [41]. These rapid and sustained effects of benralizumab in various inflammatory conditions could be attributed to its multifaceted mechanism of action, as it extends beyond NK cell-mediated eosinophil elimination, depleting other cells such as basophils and involving the recruitment and modulation of other immune cells, such as macrophages, which work together to synergistically amplify the potential of reducing eosinophilic inflammation [4,9,42,43,44,45].

In summary, our findings support the early response and sustained benefits of benralizumab, emphasizing the importance of the early identification of ESRs to personalize biologic treatment strategies and optimize clinical outcomes. The inherent limitations of our study stem from its observational and retrospective design. As a real-world study, we face common challenges, such as potential unmeasured confounders. Analyses might also be influenced by missing or partial data collection during standard clinical practice. Small subgroup sizes further limit the ability to perform formal comparisons.

## 5. Conclusions

This study highlights the early and sustained benefits of benralizumab in SEA patients, particularly in those meeting ESR criteria for ACT and FEV_1_ improvements. Thus, the identification of these patients may help anticipate lasting positive outcomes, emphasizing the importance of early response evaluation in clinical practice. The unique mechanism of action of benralizumab, combined with its ability to produce quick and lasting improvements, makes it a valuable treatment option for managing SEA and preventing disease progression.

## Figures and Tables

**Table 1:** Baseline sociodemographic and clinical characteristics of SEA patient subgroups classified according to early responses to benralizumab treatment in ACT score or pre-BD-FEV_1_ at 120 days.

	Based on ACT Response		Based on Pre-BD FEV_1_ (mL) Response	
Variables	ACT-TP n = 45	ACT-ESR n = 25	FEV_1_-TP n = 65	FEV_1_-ESR n = 38
**Sex, n (%)**				
Female	27 (60.0%)	15 (60.0%)	45 (69.2%)	24 (63.2%)
Male	18 (40.0%)	10 (40.0%)	20 (30.8%)	14 (36.8%)
**Age (years)**				
Mean (SD)	55.0 (11.9)	54.8 (13.0)	56.7 (13.4)	56.8 (13.9)
**BMI (Kg/m^2^); N ^a^**	44	25	65	38
Mean (SD)	28.2 (7.4)	27.9 (7.8)	27.8 (6.1)	27.2 (5.9)
Obese ^b^, n (%)	34 (77.3%)	19 (76.0%)	19 (29.2%)	10 (26.3%)
**Age at asthma onset (years); N ^a^**	42	25	55	34
Mean (SD)	31.9 (18.6)	32.5 (17.1)	32.7 (17.0)	35.5 (16.8)
**Peripheral BEC (cell/µL): N ^a^**	43	25	63	37
Median (IQR)	500.0 (295.0, 760.0)	650.0 (440.0, 1030.0)	475.0 (200.0, 770.0)	550.0 (300.0, 900.0)
**Total serum IgE concentration, (IU/mL); N ^a^**	36	24	55	33
Median (IQR)	91.0 (43.1, 362.3)	96.0 (41.8, 368.3)	100.0 (44.2, 365.5)	97.0 (47.0, 310.0)
**FeNO (ppb); N ^a^**	34	22	50	31
Median (IQR)	30.0 (18.0, 65.8)	46.0 (18.3, 100.0)	27.0 (16.6, 64.5)	30.0 (19.0, 65.5)
**Asthma duration (years) ^c^; N^a^**	42	25	55	34
Mean (SD)	23.2 (14.3)	22.3 (13.0)	21.7 (14.3)	20.4 (11.5)
**Allergic asthma, n (%)**	10 (22.2%)	5 (20.0%)	22.9 (13.1%)	8 (21.1%)
**Smoking history, n (%); N ^a^**	45	25	65	38
Never smoker	26 (57.8%)	19 (76.0%)	47 (72.3%)	29 (76.3%)
Former smoker	16 (35.6%)	5 (20.0%)	16 (24.6%)	8 (21.1%)
Smoker	3 (6.7%)	1 (4.0%)	2 (3.1%)	1 (2.6%)
**Comorbidities, n (%)**	42 (93.3%)	22 (88.0%)	61 (93.8%)	37 (97.4%)
CRSwNP	20 (44.4%)	14 (56.0%)	27 (41.5%)	19 (50.0%)
GERD	8 (17.8%)	6 (24.0%)	22 (33.8%)	15 (39.5%)
Osteoporosis	7 (15.6%)	4 (16.0%)	11 (16.9%)	6 (15.8%)
OSAS	4 (8.9%)	2 (8.0%)	9 (13.8%)	5 (13.2%)
HBP	2 (4.4%)	1 (4.0%)	11 (16.9%)	7 (18.4%)
Diabetes	3 (6.7%)	1 (4.0%)	5 (7.7%)	2 (5.3%)
Depression	4 (8.9%)	1 (4.0%)	6 (9.2%)	2 (5.3%)
Cataracts	2 (4.4%)	1 (4.0%)	4 (6.2%)	2 (5.3%)
**Patients with prior biologic treatment, n (%)**				
Patients with no prior biologic treatment	35 (77.8%)	20 (80.0%)	43 (66.2%)	26 (68.4%)
Patients with prior biologic treatment	10 (22.2%)	5 (20.0%)	22 (33.8%)	12 (31.6%)
Omalizumab	2 (4.4%)	1 (4.0%)	11 (16.9%)	3 (7.9%)
Mepolizumab **^d^**	8 (18.2%)	4 (16.0%)	11 (17.2%)	9 (24.3%)
Reslizumab	1 (2.2%)	1 (4.0%)	2 (3.1%)	1 (2.6%)

All values were calculated based on the total number of patients with available data (excluding missing values). The proportion of patients within each subgroup was calculated over the total number of patients with available data on ACT or pre-BD FEV_1_. ^a^ Patients with available data. ^b^ BMI ≥ 30 Kg/m^2^. ^c^ Time in years since first asthma symptoms appeared. ^d^ Missing data from one patient in the super-response subgroup. BEC, blood eosinophil count; BMI, body mass index; CRSwNP, chronic rhinosinusitis with nasal polyps; FeNO, fractional exhaled nitric oxide; FEV_1_, forced expiratory volume in 1 s; GERD, gastroesophageal reflux disease; HBP, high blood pressure; IgE, immunoglobulin E; IQR, interquartile range; OSAS, obstructive sleep apnoea syndrome; ESR, early super-responders; SD, standard deviation; TP, total population.

**Table 2:** Clinical outcomes of SEA patient subgroups classified according to responses to benralizumab treatment in ACT score or pre-BD-FEV_1_ at 120 days, at 1 year of follow-up after benralizumab initiation.

	Based on ACT Response		Based on FEV_1_ (mL) Response	
Variables	ACT-TP n = 45	ACT-ESR n = 25	FEV_1_-TP n = 65	FEV_1_-ESR n = 38
**Severe exacerbations; N ^a^**	45	25	65	38
**Baseline**				
Severe exacerbations, mean (SD)	2.4 (1.5)	2.5 (1.3)	2.7 (1.8)	2.8 (1.6)
Patients with zero exacerbations, n (%)	6 (13.3%)	2 (8.0%)	6 (9.2%)	2 (5.3%)
**1-year FUP**				
Severe exacerbations, mean (SD)	0.3 (0.8)	0.1 (0.4)	0.3 (0.8)	0.4 (0.9)
Patients with zero exacerbations, n (%)	35 (77.8%)	24 (96.0%)	52 (80.0%)	31 (81.6%)
**Patients with reduction in severe exacerbations, n (%) ^b^**	36 (92.3%)	22 (95.7%)	56 (94.9%)	34 (94.4%)
**Percentage reduction in severe exacerbations**	87.5%	96.0%	88.9%	85.7%
**ED visits; N ^a^**	45	25	65	38
**Baseline**				
ED visits, mean (SD)	0.6 (1.2)	0.5 (0.7)	0.6 (0.9)	0.7 (1.1)
Patients with no ED visits, n (%)	28 (62.2%)	15 (60.0%)	41 (63.1%)	22 (57.9%)
**1-year FUP**				
ED visits, mean (SD)	0.0 (0.1)	0.0 (0.0)	0.1 (0.4)	0.1 (0.5)
Patients with zero ED visits, n (%)	44 (97.8%)	25 (100.0%)	62 (95.4%)	36 (94.7%)
**Patients with reduction in ED visits, n (%) ^c^**	17 (100.0%)	10 (100.0%)	22 (91.7%)	14 (87.5%)
**Percentage reduction in ED visits**	100.0%	100.0%	83.3%	85.7%
**Hospitalizations; N ^a^**	45	25	65	38
**Baseline**				
Hospitalizations, mean (SD)	0.4 (0.9)	0.1 (0.3)	0.5 (0.8)	0.4 (0.7)
Patients with zero hospitalizations, n (%)	35 (77.8%)	23 (92.0%)	45 (69.2%)	27 (71.1%)
**1-year FUP**				
Hospitalizations, mean (SD)	0.0 (0.1)	0.0 (0.0)	0.0 (0.1)	0.0 (0.0)
Patients with zero hospitalizations, n (%)	44 (97.8%)	25 (100.0%)	64 (98.5%)	38 (100.0%)
**Patients with reduction in hospitalizations, n (%) ^d^**	9 (90.0%)	2 (100.0%)	19 (95.0%)	11 (100.0%)
**Percentage reduction in hospitalizations**	100.0%	100.0%	100.0%	100.0%
**ACT score**				
**Baseline, N ^a^**	45	25	54	32
ACT score, mean (SD)	13.9 (3.9)	13.0 (3.2)	13.6 (5.1)	13.2 (4.5)
Patients with ACT score < 20, n/N ^a^ (%)	40/45 (88.9%)	24/25 (96.0%)	46/54 (85.2%)	29/32 (90.6%)
**1-year FUP, N ^a^**	45	25	50	30
ACT score, mean (SD)	21.4 (4.8)	24.0 (1.4)	21.7 (4.9)	21.9 (4.6)
Patients with ACT score < 20, n/N ^a^ (%)	9/45 (20.0%)	0/25 (0.0%)	9/50 (18.0%)	6/30 (20.0%)
**Increase in ACT score, mean (SD)**	7.5 (5.2)	11.1 (3.1)	8.1 (5.6)	9.4 (5.7)
**Patients with ACT increase ≥ 3, n/N ^a^ (%)**	35/45 (77.8%)	25/25 (100.0%)	34/43 (79.1%)	23/26 (88.5%)
**Lung function**				
**Baseline; N ^a^**	43	25	65	38
Pre-BD FEV_1_ (% predicted), mean (SD)	65.9 (19.4)	64.7 (18.6)	69.7 (22.8)	68.3 (21.8)
Patients with pre-BD FEV_1_ < 80%, n (%)	31 (72.1%)	20 (80.0%)	43 (66.2%)	27 (71.1%)
Patients with pre-BD FEV_1_ ≥ 80%, n (%)	12 (27.9%)	5 (20.0%)	22 (33.8%)	11 (28.9%)
**1-year FUP; N ^a^**	35	20	64	37
Pre-BD FEV_1_ (% predicted), mean (SD)	78.0 (23.5)	83.4 (23.2)	79.8 (22.6)	86.5 (21.8)
Patients with pre-BD FEV_1_ < 80%, n (%)	17 (48.6%)	7 (35.0%)	33 (51.6%)	15 (40.5%)
Patients with pre-BD FEV_1_ ≥ 80%, n (%)	18 (51.4%)	13 (65.0%)	31 (48.4%)	22 (59.5%)
**Baseline; N ^a^**	42	25	65	38
Pre-BD FEV_1_ (mL), mean (SD)	1931.0 (789.5)	1847.2 (701.8)	1897.8 (745.6)	1905.8 (770.5)
**1-year FUP; N ^a^**	36	21	65	38
Pre-BD FEV_1_ (mL), mean (SD)	2265.3 (839.0)	2381.9 (733.0)	2191.4 (810.2)	2443.2 (840.3)
**Increment in pre-BD FEV_1_ (mL), mean (SD)**	430.0 (399.4)	620.0 (327.2)	293.5 (396.4)	537.4 (295.0)
**Patients with pre-BD FEV_1_ increment ≥ 100 mL, n (%)**	25/34 (73.5%)	20/21 (95.2%)	42/65 (64.6%)	36/38 (94.7%)
**Patients with pre-BD FEV_1_ increment ≥ 230 mL, n (%)**	20/34 (58.8%)	18/21 (85.7%)	37/65 (56.9%)	35/38 (92.1%)
**Patients with pre-BD FEV_1_ increment ≥ 500 mL, n (%)**	16/34 (47.1%)	15/21 (71.4%)	21/65 (32.3%)	21/38 (55.3%)
**OCS-dependency**				
**Baseline**				
OCS-dependent patients, n/N ^a^ (%)	12/44 (27.3%)	6/24 (25.0%)	18/62 (29.0%)	10/36 (27.8%)
Daily OCS dose (mg,) median (IQR)	13.0 (5.0, 22.1)	8.0 (5.0, 22.5)	15.0 (8.1, 27.7)	15.0 (6.3, 31.2)
**1-year FUP**				
OCS-dependent patients, n/N ^a^ (%)	7/45 (15.6%)	2/25 (8.0%)	11/65 (16.9%)	5/38 (13.2%)
Daily OCS dose (mg,) median (IQR) ^e^	3.0 (0.0, 10.4)	0.0 (0.0, 0.0)	0.0 (0.0, 9.6)	0.0 (0.0, 0.0)
**Patients with OCS dose reduction ≥ 50%, n/N ^a^ (%)**	7/12 (58.3%)	5/6 (83.3%)	13/18 (72.2%)	8/10 (80.0%)
**Patients achieving complete OCS withdrawal, n/N ^a^ (%)**	6/12 (50.0%)	5/6 (83.3%)	10/18 (55.6%)	8/10 (80.0%)
**Percentage reduction in median daily OCS dose**	76.9%	100.0%	100.0%	100.0%

All values were calculated based on the total number of patients with available data (excluding missing values). ^a^ Patients with available data. ^b^ Proportion of patients with 1 or more exacerbations at baseline who achieved a decrease in the frequency of exacerbations at 1-year FUP. ^c, d^ Calculations were made over the total number of patients with ED visits or hospitalizations, respectively. ^e^ Median daily dose of OCS at 1-year is estimated on OCS-dependent patients at baseline. ACT, asthma control test; ED, emergency department; FEV_1_, forced expiratory volume in 1 s; FUP, follow-up; pre-BD, pre-bronchodilator; OCS, oral corticosteroids; ESR, early super-responders; SD, standard deviation; TP, total population.

## Data Availability

The datasets used and analysed during the current study may be obtained in accordance with AstraZeneca’s data sharing policy, described at: https://astrazenecagrouptrials.pharmacm.com/ST/Submission/Disclosure, accessed on 10 February 2025.

## Abbreviations

The following abbreviations are used in this manuscript:

ACT	Asthma control test
FUP	Follow-up
NER	Non-early responders
OCS	Oral corticosteroids
FeNO	Fractional exhaled nitric oxide
IgE	Immunoglobulin E
Pre-BD FEV_1_	Pre-bronchodilator forced expiratory volume in 1 second
ER	Early responders
ESR	Early super-responders
SEA	Severe eosinophilic asthma

## Appendix A

**Investigators and sites**

Alicia Padilla Galo and Borja Valencia Azcona (Hospital Costa del Sol, Málaga); José Luis Velasco Garrido, Álvaro Martínez Mesa, Alberto Levy Naón, Isabel Moya Carmona and Carmen Cañete Luque (Hospital Virgen de la Victoria, Málaga); Ismael García Moguel and Rocío Díaz Campos (Hospital Universitario 12 de Octubre, Madrid); Pilar Ausín and Eugenia Navarrete (Hospital del Mar, Barcelona); Luis Carazo Fernández (Complejo Asistencial Universitario de León, León); Eva Martínez Moragón (Hospital Universitario Doctor Peset, Valencia); Carlos Martínez Rivera (Hospital Universitario Germans Trias i Pujol, Barcelona); Francisco Casas Maldonado and Luis F. Cassini Gómez de Cádiz (Hospital San Cecilio, Granada); Rubén Andújar Espinosa (Hospital Clínico Universitario Virgen de la Arrixaca, Murcia); Elisabeth Vera Solsona and Marta Forner Vicente (Hospital Universitario Miguel Servet, Zaragoza); Andrea Trisán Alonso (Hospital Universitario Puerta de Hierro, Madrid); Fernando Sánchez-Toril López (Hospital Arnau de Vilanova, Valencia); Marcela Valverde Monge and Erwin Javier Pinillos (Hospital Universitario Fundación Jiménez Díaz, Madrid); Marina Blanco Aparicio (Complejo Hospitalario Universitario A Coruña, La Coruña); Marta Palop Cervera (Hospital de Sagunto, Valencia).
